# Osmotic Dehydration of Japanese Quince (*Chaenomeles japonica*) Fruits in Erythritol Solutions: Impact of Processing Conditions on the Kinetic Parameters and on Physicochemical and Antioxidant Properties of the Fruits

**DOI:** 10.3390/molecules29235524

**Published:** 2024-11-22

**Authors:** Angelika Wojtyś, Sławomir Pietrzyk, Sylwia Bogacz, Robert Witkowicz

**Affiliations:** 1 Department of Food Analysis and Evaluation of Food Quality, Faculty of Food Technology, University of Agriculture in Krakow, Balicka 122, 30-149 Krakow, Polandsylwia.bogacz@student.urk.edu.pl (S.B.); 2 Department of Agroecology and Crop Production, University of Agriculture in Krakow, Mickiewicza 21, 31-120 Krakow, Poland

**Keywords:** Japanese quince, osmotic dehydration, erythritol, sugar profile, antioxidant properties

## Abstract

The present work aimed to evaluate the effectiveness of erythritol as an osmotic agent in the osmotic dehydration (OD) process of Japanese quince fruits and to assess its effects on their physicochemical and antioxidant properties. The efficiency of the OD process was determined by examining its kinetics and comparing the results to those from a sucrose solution. In selected osmotically dehydrated fruits, the following parameters were determined: dry matter content, total acidity, pH, sugar profile, color parameters, total phenolic and flavonoid content, antioxidant activity (DPPH and ABTS assays), and vitamin C content. Moreover, principal component analysis (PCA) was also performed. Generally, the OD process using a 40% erythritol solution demonstrated the most efficient kinetics among all osmotic agents tested; however, fruits dehydrated in this solution also showed the most significant changes in physicochemical and antioxidant properties compared to fresh fruits. Remarkably, Japanese quince fruits dehydrated in a 30% erythritol solution exhibited higher levels of total phenolic, flavonoid, vitamin C, and antioxidant activity than those treated with a 50% sucrose solution, despite displaying similar dehydration kinetics. The use of erythritol solutions also led to a decrease in sugar content. In turn, PCA analysis confirmed a correlation between pH values and the L* color parameter, with the highest L* value observed in fruits dehydrated with the sucrose solution. Thus, erythritol may be utilized as a low-calorie alternative to sucrose as an osmotic agent while simultaneously minimizing the loss of antioxidant compounds.

## 1. Introduction

Japanese quince (*Chaenomeles japonica*) fruits are recognized as a rich source of bioactive compounds with numerous health benefits. These fruits are particularly valued for their low sugar content, high dietary fiber, and significant amounts of vitamin C, organic acids (mainly malic, succinic, and quinic acids), and essential minerals (potassium, magnesium, calcium, iron, zinc, manganese, and copper). Japanese quince is also a notable source of polyphenols, including flavonoids and phenolic acids, with dominant compounds like flavanols and catechins. Additionally, the fruits contain quercetin glycosides, chlorogenic acid, and triterpenes, including ursolic and oleanolic acids. However, the high concentrations of organic acids and phenolic compounds contribute to the fruit’s distinctly sour and astringent taste, making it less suitable for raw consumption. As a result, these fruits are predominantly used in the industrial production of juices, jams, jellies, liquors, and wines [1,2,3].

In recent years, there has been growing interest in the application of osmotic dehydration (OD) to produce functional and/or low-calorie foods, which could be consumed directly as snacks (e.g., fruit chips) or used as ingredients in various food products such as dairy products, breakfast cereals, tea mixes, or confectionery. Osmotic dehydration is a gentle method of removing water from plant-based products without phase change. It effectively reduces water activity in fruits, yielding final products that maintain nutritional and sensory attributes—including color, texture, taste, and aroma—comparable to those of fresh fruit. Additionally, this process offers several advantages, such as improved product quality and reduced energy consumption [4]. Osmotic dehydration entails the immersion of plant tissue in a hypertonic solution, often a sugar-based solution, generating an osmotic pressure gradient that facilitates counter-current mass transfer. During this process, water is extracted from the plant material into the osmotic solution, while the solutes from the solution diffuse into the plant tissue. However, the semipermeable nature of the cell membrane can lead to the concurrent loss of valuable nutrients, including vitamins, minerals, antioxidants, pigments, and aromatic compounds, during water removal [5,6]. To optimize the osmotic dehydration process, it is crucial to select appropriate parameters that minimize the absorption of soluble solids from the osmotic solution while maximizing the retention of the plant material’s nutrients. The effectiveness of the OD process is influenced by several factors, including temperature and duration of the process, the structure and geometry of the raw material (shape, size), the concentration and type of osmotic solution, and the ratio of material mass to osmotic solution mass [7,8].

The choice of an appropriate osmotic solution is crucial for the effective osmotic dehydration of plant materials. Generally, the osmotic solution should be non-toxic and highly soluble in low-molecular-weight substances, facilitating the efficient reduction in water content and activity in the raw material [9,10]. Sucrose is the most widely used osmotic agent in fruit processing due to its availability, efficiency, and cost-effectiveness. There are many studies concerning using sucrose as an osmotic solution in the osmotic dehydration of apple [11], apricot, chokeberry [12], kiwifruit [13], cranberry [14], and pears [15]. In addition to sucrose, various studies have examined the effects of other sugars, such as glucose, fructose, maltose, oligofructose, and trehalose, on the OD process [16]. Recent trends in OD, however, are shifting towards the use of alternative osmotic agents, particularly those with a low glycemic index, such as polyols [10]. Research has explored the use of polyols like sorbitol [10,17], erythritol, glicerol [17], maltitol [10], and xylitol [18] as substitutes for sucrose solutions in the osmotic dehydration of various fruits. 

Erythritol, a sugar alcohol composed of four carbon atoms, is one of the simplest polyols, resulting in a relatively low molecular mass compared to other polyols. This lower molecular weight is associated with distinct physico-chemical properties, such as its lower viscosity and high solubility, which differentiate it from other polyols. It exhibits moderate sweetness (60–80% of sucrose), lacks a significant aftertaste, and can mask undesirable tastes, such as astringency, while improving mouthfeel. Due to its high negative heat of solution, erythritol also produces a noticeable cooling effect when dissolved. Importantly, erythritol is non-caloric, as it is poorly absorbed by the body and excreted unchanged in urine, without affecting blood glucose or insulin levels. While other polyols are commonly associated with digestive discomfort, erythritol is notably well tolerated due to its relatively small molecular size. Additionally, erythritol has antioxidant properties, acting as a free radical scavenger in the body before excretion [19]. 

Despite this growing interest, there are still few studies specifically focusing on the effects of erythritol in the OD process, and to date, there is no available research on how these solutions impact the antioxidant properties of osmo-dehydrated fruits. Furthermore, previously published studies have predominantly focused on the osmotic dehydration of more popular and commercially available fruits (e.g., apples, strawberries). Japanese quince fruits, with their distinct physical characteristics (very hard fruits unsuitable for direct consumption) and exceptional nutritional composition (high levels of vitamin C, antioxidants, fiber, and essential minerals), enhance research with a new focus. Their unique properties may result in the osmotic dehydration process behaving differently compared to commonly osmotically dehydrated fruits. Thus, the aim of this work was to assess the effectiveness of erythritol as an osmotic agent compared to sucrose solution, and then to evaluate the changes in physicochemical and antioxidant properties of osmotically dehydrated Japanese quince fruits. 

## 2. Results and Discussion

### 2.1. Kinetic Parameters of the Osmotic Dehydration Process

Osmotic dehydration involves a bidirectional mass transfer process: water is extracted from the material into a hypertonic solution, while various solutes from the osmotic solution diffuse into the material, which has a lower osmotic potential [20]. To evaluate mass transfer during the osmotic dehydration (OD) process of Japanese quince fruits, the following parameters were determined: water loss (WL), mass loss (ML), and solids gain (SG).

The kinetics of the quince fruits’ osmotic dehydration process depends on all the experimental factors studied (solution, temperature, and time), as confirmed by the table evaluating the impact of the main effects (Table 1).

**Figure 1:**
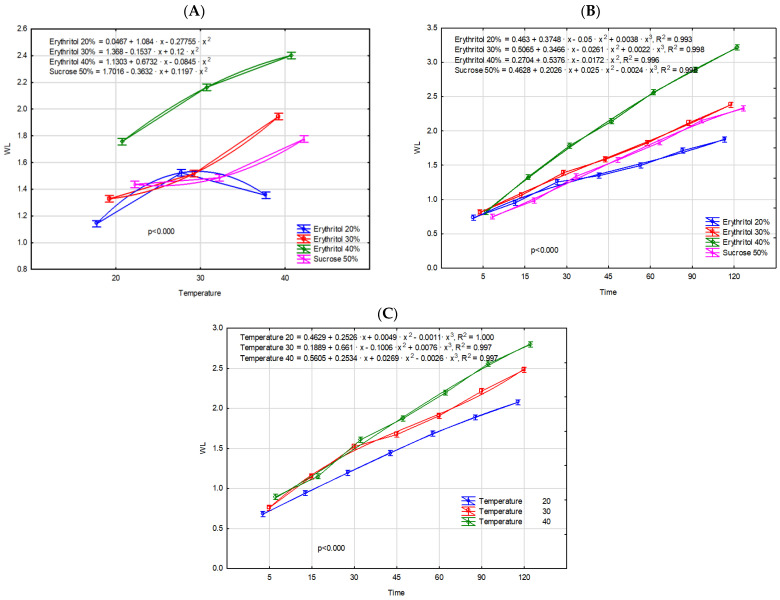
Water loss [g H_2_O/g i.d.m.] during OD process of Japanese quince fruits as a result of interaction of experimental factors: (**A**)—temperature × type of osmotic solution, (**B**)—time × type of osmotic solution, (**C**)—time × temperature.

The water loss progressed with increasing temperature and dehydration time, following polynomial functions characterized by a decrease in the rate of water loss as the process advanced. Moreover, the water loss of the fruits at specific evaluation points (temperature increase and time progression) demonstrated statistically significant differences. The influence of temperature on the OD process was also confirmed by Devic et al. [21] during the osmo-dehydration of apples at 45 °C and 60 °C, and Ferrari and Hubinger [22] in osmotically dehydrated melon cubes at 30 °C and 40 °C. An increase in temperature reduces the viscosity of the osmotic solution, thereby lowering the external resistance to mass transfer between the sample surface and the osmotic solution, which facilitates water loss from the fruits and increases the diffusion rate of the solute into the sample. The Japanese quince fruits exhibited a slightly different dehydration pattern in response to increasing erythritol concentration, as the intensity of the process increased linearly with rising erythritol concentration. The effects of the interaction of the research factors on the water loss process are presented in Figure 1. Briefly, higher concentrations of the osmotic solution result in a higher osmotic driving force between the plant tissue and the surrounding osmotic solution [20]. The obtained results are in accordance with those reported by Cichowska et al. [16] and Wiktor et al. [10], who investigated the impact of osmotic agent concentration on water mass loss during the osmotic dehydration of apples in erythritol, xylitol, and maltitol solutions (at concentrations of 20–50%), as well as organic strawberries in 50% sucrose, 20–30% maltitol, and 20–40% sorbitol solutions. The dehydration process of quince fruits increases linearly with increasing temperature solely in the case of erythritol solutions at concentrations of 30% and 40%. Nevertheless, the 40% concentration enables significantly more effective dehydration at 30 °C and 40 °C compared to the other solutions used, including the 50% sucrose solution (Figure 1A). The nonlinearity with respect to temperature in a 20% erythritol solution is likely related to the insufficient osmotic gradient generated by the low concentration of the osmotic solution. At a temperature of 40 ºC, cell membranes are less stable and less selective during osmotic dehydration Moreover air bubbles trapped in the intercellular spaces more intensive migrate to the osmotic solution. These processes may reduce the effectiveness of osmotic dehydration in low concentrations of erythritol.

**Figure 2:**
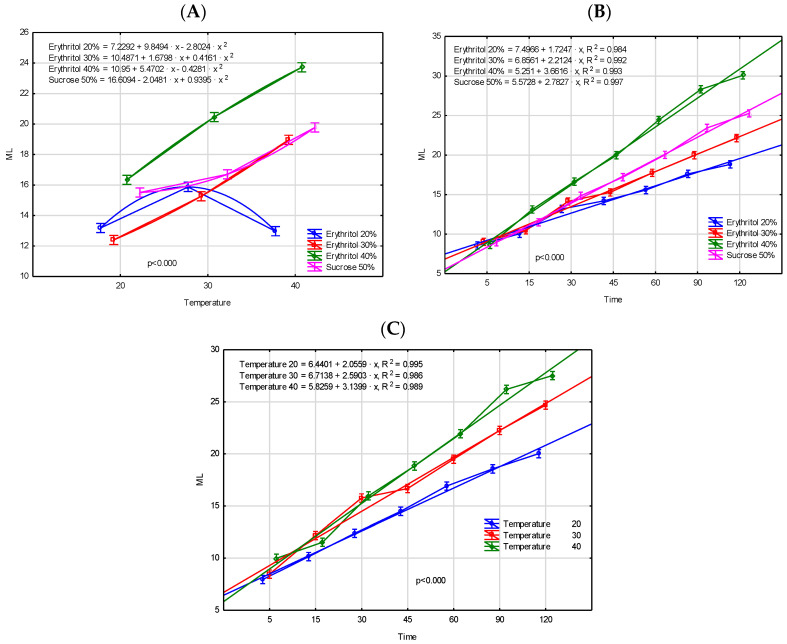
Mass loss [%] during OD process of Japanese quince fruits as a result of interaction of experimental factors: (**A**)—temperature × type of osmotic solution, (**B**)—time × type of osmotic solution, (**C**)—time × temperature.

It can therefore be concluded that erythritol is a more effective osmotic solution (even at a lower concentration) compared to sucrose. This is attributed to the differences in molecular weight between erythritol and sucrose, which are 122.12 g/mol and 342.30 g/mol, respectively. The lower molecular weight of the solute enhances the driving force of the osmotic dehydration process, leading to more rapid mass transfer, as reflected in the effective diffusivity of water or solids. While the concentration of the solution is widely recognized as a key factor influencing the efficiency of osmotic dehydration, the solute’s molecular size also plays a crucial role in determining the rates of water loss and solid gain [20]. Furthermore, the use of a higher sucrose concentration may lead to tissue hardening, thereby inhibiting mass transport in the dehydrated fruits [22]. Similar results were observed by Cichowska et al. [16]. Figure 1B further demonstrates the superiority of the 40% erythritol solution over the other solutions as early as the 15 min mark. In evaluating the impact of the interaction between time and temperature on the water loss parameters, it is important to highlight the 45 min mark, which represents a point of distinct differentiation in the dynamics of this process depending on temperature (Figure 1C). Since WL and ML parameters are mathematically equivalent processes, as confirmed by the observed correlation (r = 0.979), they are presented below but not discussed in detail (Table 1, Figure 2).

**Figure 3:**
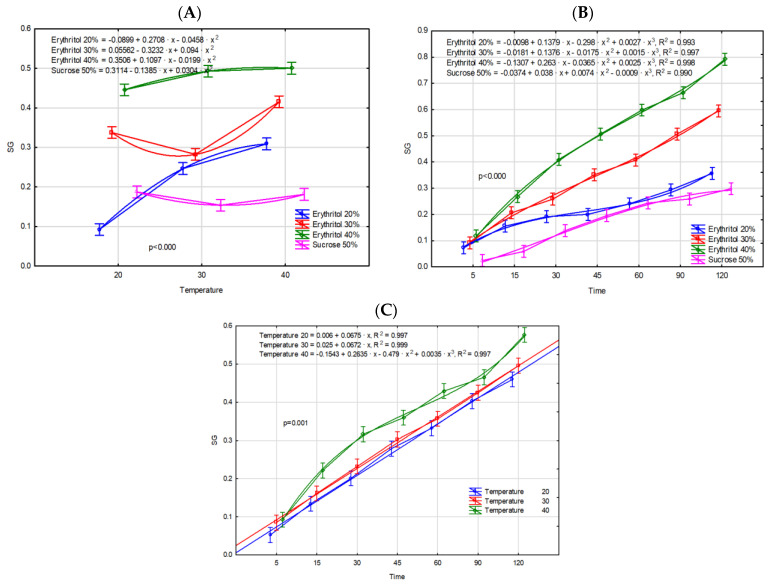
Solids gain [g d.m./g i.d.m] during OD process of Japanese quince fruits as a result of interaction of experimental factors: (**A**)—temperature × type of osmotic solution, (**B**)—time × type of osmotic solution, (**C**)—time × temperature.

Due to the moderately high correlation coefficient between water loss and solids gain (0.742), a distinct difference in the influence of the experimental factors on the kinetics of solids gain was observed. SG is a crucial parameter that reflects the quantity of soluble solids absorbed by the sample during osmotic dehydration. The effective OD process is characterized by significant water loss accompanied by minimal solids gain [23]. The lowest increase in solids gain was recorded using the 50% sucrose solution. In contrast, all applied erythritol concentrations resulted in a statistically significant increase in solids gain. The variations in SG values observed for different osmotic agents can be attributed to their molecular sizes. Solutes with higher molecular weights tend to remain on the surface of the tissue, leading to lower SG values. Conversely, smaller molecules can diffuse more readily through the product matrix, enhancing the solids gain [24]. Therefore, erythritol, which has a lower molecular weight than sucrose, diffuses more easily through the cells, facilitating an increase in SG. Moreover, higher-concentration solutions exhibit increased viscosity, which can impede sugar uptake by promoting the formation of a superficial solute layer around the fruit [21]. Comparable results have been reported previously for other food matrices subjected to the osmotic dehydration process in different solutions [16,17,18,25]. For the remaining factors, specifically temperature and time, values increased proportionally with higher experimental levels (Table 1). The interaction of solution × temperature (Figure 3A) clearly reveals that the highest SG parameter values were attained across all temperature conditions with the application of the 40% erythritol solution. Furthermore, applying a temperature of 40 °C seems unwarranted, as a 40% erythritol solution at 30 °C yields a comparable solids gain. The evaluation of the interaction of solution × time (Figure 3B) corroborates that the 40% erythritol solution demonstrates the most pronounced dynamics in the increase in solids gain, whereas the sucrose solution exhibited the lowest dynamics. In turn, the interaction temperature x time demonstrates that at 20 °C and 30 °C, the solids gain was comparable and exhibited less variability compared to the more dynamic changes observed at 40 °C. According to Abbasi-Souraki et al. [26], increasing the temperature of the process enhances the mobility of water molecules along with the dissolved substances. Furthermore, at higher temperatures, the viscosity of the osmotic solution decreases, and the plant cell membranes become plasticized, swollen, or disrupted, which may influence the kinetics of solids gain.

Based on the presented results, it can be clearly stated that the use of a 40% erythritol solution ensures the most efficient dehydration of the raw material (Table 1, Figure 3B). The most effective temperature, providing the greatest dehydration dynamics over time, is 40 °C (Table 1, Figure 3C). Erythritol solutions (both 30% and 40%) dehydrated more effectively at all temperatures than the use of a 50% sucrose solution and a 20% erythritol solution (Figure 3B). When seeking the best combination of solution × temperature, it can be definitively stated that the optimal combination is 40% erythritol × 40 °C. It should be emphasized that the curves fitted to the dehydration process in the combinations of 20% and 30% erythritol in relation to temperature have a different shape than the model observed for the 40% erythritol solution. Of course, the distinct shapes of the models are confirmed by the equation parameters shown (Figure 3A). Therefore, further analyses were conducted exclusively on samples osmotically dehydrated in 30% and 40% erythritol solutions at temperature of 40 °C, as well as on fresh fruits.

### 2.2. Physicochemical and Antioxidant Properties of Osmotically Dehydrated Japanese Quince Fruits

#### 2.2.1. Effect of Osmotic Dehydration Process on Dry Matter Content, Total Acidity, pH, and Sugar Profile of Japanese Quince Fruits

The fresh Japanese quince fruits were characterized by a dry matter content of 12.39% (Table 2). A similar result was observed by Tarko et al. [27], while Urbanavičiūtė and Viškelis [28] reported lower dry matter values. In turn, Turkiewicz et al. [29], who also investigated various cultivars of Japanese quince, found that dry matter content in fresh fruits to even up to 20%. The differences in the dry matter content of fresh quince fruits may depend on various aspects. The primary factor influencing dry matter content is sun exposure, with insufficient sunlight leading to lower dry matter. Other important factors include the stage of maturity, cultivar type, climatic conditions, and agrotechnical practices [27]. The osmo-dehydration process resulted in an increased in dry matter content, regardless of the osmotic solution used. Erythritol was found to be a more effective osmotic solution compared to sucrose solution. The dry matter values for fruits dehydrated in 30% and 40% erythritol solutions were 2.5 and 3 times higher, respectively, than in fresh fruits, whereas for the sucrose solution, the increase was 2-fold. Higher values of dry matter in osmo-dehydrated fruits compared to fresh fruits were mainly caused by the penetration of soluble solids from the osmotic solution into the plant tissue [30]. 

Japanese quince is commonly known for its high acidity and low sugar content, which makes it unsuitable for raw consumption. In our study, the total acidity (as malic acid) of analyzed fresh fruits was 4.27% (Table 2). This is in accordance with the results obtained by Turkiewicz et al. [29] and Ros et al. [31]. During osmotic dehydration, a significant quantity of acidic compounds migrated from the fruit tissue into the osmotic solution. The greatest reduction in total acidity, compared to fresh Japanese quince, was observed when a 50% sucrose solution was used. For erythritol, a higher concentration of the solution led to a slightly greater decrease in fruit acidity, although the differences were minimal. Specifically, a 40% erythritol solution reduced acidity more effectively than a 30% erythritol solution when compared to fresh fruits. The high acidity of Japanese quince fruit is associated with a low pH value. According to the literature [31], the pH value of these fruits, depending on the variety and ripening conditions, ranges from 2.40 to 2.99, which is consistent with the results obtained in this study. The osmotic dehydration process caused a significant increase in pH in all analyzed samples. Statistical analysis showed that varying erythritol concentrations in the osmotic solution did not significantly affect pH values. Only the type of osmotic solution used had a significant effect on the final values of this parameter. Studies conducted by Khoualdia et al. [32] and Fasogbon et al. [33] on the dehydration of pomegranate peel and pineapple slices in sucrose solution, respectively, confirm the migration of acidic substances into the solution during osmotic dehydration. Furthermore, Khoualdia et al. [32] demonstrated that a significant decrease in acidity, and consequently an increase in pH, occurs within the first 30 min of the process.

The sugar profile of fresh Japanese quince was characterized mainly by a glucose content of 11.87 g/100 g dry matter (d.m.) and fructose content of 9.23 g/100 g d.m., which corresponds to 1.47 g and 1.11 g per 100 g of fruit, respectively (Table 2). Similar results were obtained by Tarko et al. [27]. In turn, Turkiewicz et al. [29] analyzed different species and cultivars of quince fruits and found that sugar content in fresh fruits ranged from 0.43 to 3.98 g/100 g fresh weight. The proportion of sugars in fresh fruits may vary, depending on various factors such as quince variety, stage of ripeness, and growing conditions. Aksic et al. [34] explains that the variability in sugar metabolism plays a critical role in modulating plant responses to abiotic stress. Under stressful conditions, plants dynamically adjust sugar metabolism, where sugars act as signaling molecules, activating stress-responsive pathways that enhance the plant’s ability to adapt and persist in adverse environmental conditions. Osmotic dehydration in both erythritol and sucrose solutions resulted in significant changes in the sugar profile of Japanese quince. The OD process in the erythritol solution led to the loss of nearly 90% of sugars, including both glucose and fructose. The content of simple sugars in fruits osmo-dehydrated in different concentrations of erythritol solutions did not differ significantly. Furthermore, during osmotic dehydration, some erythritol molecules diffused from the solution into the fruit tissue. In fruits dehydrated in a 30% erythritol solution, the polyol content was 42.90 g/100 g d.m., whereas a higher concentration of the solution resulted in 1.5 times higher erythritol levels, reaching 63.07 g/100 g d.m. Essentially, the sugars in plant tissue were almost completely replaced by erythritol molecules. This finding is consistent with results reported by Wiktor et al. [10], who analyzed strawberries subjected to osmotic dehydration in various polyol solutions. In contrast, the use of a 50% sucrose solution did not cause losses in glucose and fructose in quince fruits as significant as those observed with erythritol solutions. The glucose and fructose contents in the dehydrated fruits were approximately 35% and 17% lower, respectively, compared to fresh quince fruits. This phenomenon can be explained by differences in osmotic pressure between fruit tissue and osmotic solution, which drive the process. Moreover, during the osmotic dehydration of fruit at higher temperatures and in an acidic environment (high acidity of the fruit), sucrose is likely hydrolyzed into simpler sugars, which then further penetrate the plant tissue, thereby increasing the content of fructose and glucose in the sample. Consistent conclusions were reached by Delgado et al. [35], who osmotically dehydrated chestnut slices in sucrose solutions. During the OD process, sucrose molecules penetrated into the plant tissues, with values comparable to those achieved using a 30% erythritol solution. Analyzing the obtained sugar contents and mass transport values (Figure 1, Figure 2 and Figure 3) in dehydrated quince fruit, it can be concluded that the use of a 30% erythritol solution enables the production of a final product with a sweet taste (erythritol’s sweetness is approximately 80% that of sucrose), while also reducing total sugar content and thus lowering the caloric value [19]. It should also be noted that excessive consumption of polyols, including erythritol, can cause undesirable intestinal gastrointestinal effects, such as bloating and abdominal cramps, or a laxative effect. However, Tetzloff et al. [36] demonstrated that repeated ingestion of erythritol at a daily dose of 1 g/kg body weight is well tolerated by the body. Cichowska-Bogusz et al. [37] conducted calculations demonstrating that apples subjected to dehydration in erythritol solutions followed by drying did not exhibit polyol concentrations at levels sufficient to induce gastrointestinal disturbances. Comparing the results obtained in our study with those of Cichowska-Bogusz et al. [37], similar conclusions can be drawn.

#### 2.2.2. Effect of Osmotic Dehydration Process on Total Phenolic Content, Total Flavonoids Content, and Vitamin C in Japanese Quince Fruits

Japanese quince fruits are widely regarded as a rich source of polyphenolic compounds, with flavonoids being particularly significant. According to the literature, they exhibit the highest levels of polyphenolic compounds compared to various stone fruits such as apples, pears, nectarines, plums, cherries, and peaches [3]. The total flavonoid content (TFC) and the total phenolic content (TPC) in Japanese quince, fresh and subjected to osmotic dehydration in sucrose and erythritol solutions, are presented in Table 3. The total phenolic content in analyzed fresh fruits was 228.63 mg GAE/g d.m, which refers to 28.33 mg GAE/g f.w. The obtained results were similar to those presented by Kikowska et al. [38]. In turn, the content of total flavonoids in fresh Japanese quince fruits was 28.41 mg QE/g d.m., which corresponds to 3.52 mg QE/g f.w. 

As expected, osmotic dehydration, as well as the type of osmotic solution used in the process, caused a significant decrease in both the total phenolic content and the total flavonoid content of Japanese quince fruits. The most pronounced reduction in TPC, approximately 85%, was observed in fruits subjected to osmotic dehydration in a 40% erythritol solution. Conversely, a lower erythritol concentration (30%) resulted in the smallest decline in TPC, with a reduction of 80%. The use of a 50% sucrose solution resulted in a reduction in total phenolic content by approximately 82%. Similar trends were demonstrated for TFC, with losses of these compounds amounting to 70%, 60%, and 65%, respectively. The decrease in TPC in osmotically dehydrated quince fruits was likely partly due to the reduction in TFC. Notably, osmotic dehydration of quince fruit in a lower concentration of erythritol (30%) led to smaller losses of phenolic compounds compared to the 50% sucrose solution, despite the OD processes exhibiting similar kinetics (Figure 1, Figure 2 and Figure 3). These results indicate that not only the concentration of the osmotic solution, but primarily its type, significantly affected the loss of phenolic compounds. Additionally, a strong negative correlation was demonstrated between the parameters ML and WL and the total phenolic content (ML: r = −0.992; WL: r = −0.978) as well as the total flavonoid content (ML: r = −0.998; WL: r = −0.984). Yu et al. [39] analyzed the total phenolic content in both osmotically dehydrated blueberries and the osmotic solution after OD process. Their study confirmed that a significant quantity of polyphenolic compounds (approximately 78%) migrates from the fruit tissues into the liquid medium during osmotic dehydration, resulting in lower concentrations in the dehydrated fruits and higher concentrations in the solution. Moreover, the degradation of polyphenolic compounds is further attributed to oxidative and/or hydrolytic processes that certain polyphenols undergo. During osmotic dehydration, the fruits are subjected to prolonged immersion at a temperature near the optimal activity range of polyphenol oxidase (40 °C), an enzyme responsible for the degradation of a broad spectrum of phenolic compounds, including flavonoids and phenolic acids [40]. The decrease in TPC, as well as TFC values during the osmotic dehydration of fruits in various osmotic solutions, have also been corroborated by other studies [10,39,40,41,42,43].

The amount of vitamin C (ascorbic acid) in fresh Japanese quince fruits was 4.81 mg/g d.m., corresponding to 0.60 mg/g in fresh fruits (Table 3). Similar vitamin C content in Japanese quince was reported by Tang et al. [44] and Hallmann et al. [45], at 0.64 and 0.63 mg/g of f.w., respectively. Osmotic dehydration caused a significant reduction in vitamin C content, irrespective of the type of osmotic solution used. Following the OD process in erythritol solutions, ascorbic acid losses amounted to approximately 85% compared to fresh Japanese quince fruits. Samples osmotically dehydrated in 30% and 40% erythritol solutions did not exhibit significant differences in vitamin C reduction. The OD process in a 50% sucrose solution resulted in a slightly lower loss of this compound, around 75%. Similarly, Araya-Farias et al. [46] observed a 78% decrease in vitamin C content during the osmotic dehydration of seabuckthorn fruits in sucrose solutions. In contrast, Sakooei-Vayghan et al. [41] reported a 25% reduction in vitamin C in osmotically dehydrated apricot cubes. Generally, the loss of vitamin C during the OD process is primarily attributed to its water solubility and subsequent leakage into the osmotic solutions. However, considering the results of vitamin C content, as well as those obtained from the mass exchange, it can be noted that the loss of vitamin C may be caused not only by the reduction in the osmotic pressure gradient during osmotic dehydration, but also by many other factors. Wiktor et al. [10], while examining the effect of various osmotic solutions (sorbitol, mannitol, and sucrose) on the vitamin C content in organic strawberries, indicated that different concentrations of individual osmotic agents can induce varying structural changes in fruit tissues, thereby leading to different levels of vitamin C in the dehydrated fruits. The authors reported that the vitamin C content after osmotic dehydration ranged between 22% and 43% of the levels found in fresh fruit, depending on the osmotic agent used.

The antioxidant activity (AA) of the analyzed fresh Japanese quince fruits was similar to that reported by Turkiewicz et al. [29]; however, lower antioxidant activity values were observed in the studies conducted by Tarko et al. [27]. The antioxidant activity of fruits is influenced by various factors, including variety, genotype, fruit ripeness, growing season, storage duration after harvest, and processing methods [14].

The osmotic dehydration process resulted in a significant decrease in the antioxidant activity of Japanese quince fruits, as measured by both DPPH and ABTS assays (Table 3). Wiktor et al. [10] reported lower antioxidant activity in organic strawberries dehydrated in sucrose, sorbitol, and mannitol solutions, while Yu et al. [39] observed similar findings in blueberries dehydrated in sucrose solution. In turn, Kowalska et al. [40] showed that the OD process of strawberries in sucrose and inulin solution can enhance their antioxidant activity compared to fresh fruits. In all analyzed fruits, the ABTS assay showed higher AA values. Although both techniques used to assess antioxidant activity are based on electron transfer [10], the differences in the obtained results could be influenced by various mechanisms of scavenging of these two free radicals (such as reagent concentration, incubation time, or differences in reaction kinetics). The reduction in antioxidant activity in the all investigated samples ranged from 60% to 75% for DPPH, and 60% to 70% for ABTS, compared to fresh fruits. Similar to the results for TPC and TFC, the most substantial decreases in AA were observed in fruits subjected to the OD process in a 40% erythritol solution, while the lowest, though still significant, impact on AA was found in those treated with a 30% erythritol solution. Statistical analysis showed a high and significant linear correlation between TPC, TFC, and vitamin C content with antioxidant activity, as measured by both DPPH (TPC: r = 0.981; TFC: r = 0.987, vitamin C: r = 0.941) and ABTS (TPC: r = 0.997; TFC: r = 0.999, vitamin C: r = 0.979) assays. That results confirmed that polyphenols and vitamin C are among the primary antioxidants present in Japanese quince fruits. Furthermore, Pearson’s correlation analysis revealed a strong negative correlation between the parameters ML and WL and both DPPH (ML: r = −0.983; WL: r = −0.956) and ABTS (ML: r = −0.996; WL: r = −0.983) assays.

Overall, Japanese quince fruits dehydrated in a 30% erythritol solution exhibited higher levels of TPC, TFC, and vitamin C, as well as improved antioxidant activity, compared to those subjected to a traditional sucrose solution, despite showing similar dehydration kinetics. This suggests that using erythritol at a lower concentration could be a better alternative as an osmotic agent during the dehydration process.

#### 2.2.3. Effect of Osmotic Dehydration Process on Color Parameters of Japanese Quince Fruits

Color is one of the key quality parameters in fruit products and is the primary factor customers use to assess product quality. It is crucial to monitor color changes during osmotic dehydration, as color loss is one of the most significant alterations in this process. The Japanese quince fruits were characterized by L*, a*, and b* parameters of 47.92, 2.37, and 22.71, respectively. The osmotic dehydration process of Japanese quince in both erythritol and sucrose solutions resulted in significant changes in color parameters compared to fresh fruits (Table 4). 

All osmo-dehydrated fruits were characterized by higher L* values, indicating increased lightness of the sample. The most substantial changes were observed in fruits osmo-dehydrated in a 50% sucrose solution, while the smallest were observed in the case of 30% erythritol solution. The increase in L* values in osmo-dehydrated Japanese quince fruits could be due to osmotic solution molecules penetrating the fruit tissue, forming a more solid structure. The crystalline structure of sucrose and erythritol tends to reflect light better than the water-filled structure, further increasing the L* values. According to Krokida et al. [47], osmotic dehydration can also reduce water activity, which in turn leads to a decrease in non-enzymatic browning reactions. The highest L* values in fruits dehydrated in a 50% sucrose solution were probably due to solution accumulation on the fruit tissue surface, resulting from increased absorption at higher concentrations [48]. The OD process caused a significant decrease in both the a* parameter (describing redness) and the b* parameter (describing yellowness) in almost all analyzed samples, except for fruits osmo-dehydrated in a 50% sucrose solution, in which no significant differences in the b* parameter were observed. Moreover, the a* parameter showed a significant negative correlation with the ML and WL parameters (ML: r = −0.888; WL: r = −0.884). This suggests that during the osmo-dehydration process, some amount of the fruit’s colorants could be lost, thereby leading to changes in color. Based on the total color difference (ΔE) values, it was found that the color of Japanese quince fruits subjected to osmotic dehydration differed visibly from that of fresh fruits. The obtained ΔE values ranged from 6.17 (for 30% erythritol solution) to 13.83 (for 50% sucrose solution). According to Mokrzycki and Tatol [49], this indicates that the color change between the analyzed samples can be perceived by an observer as two distinct colors (ΔE > 5). The impact of the OD process on color alterations in fruits dehydrated in sucrose and polyol solutions (e.g., xylitol, sorbitol, mannitol, and erythritol) has also been reported by other authors [10,15,30]. 

### 2.3. Principal Component Analysis

**Figure 4:**
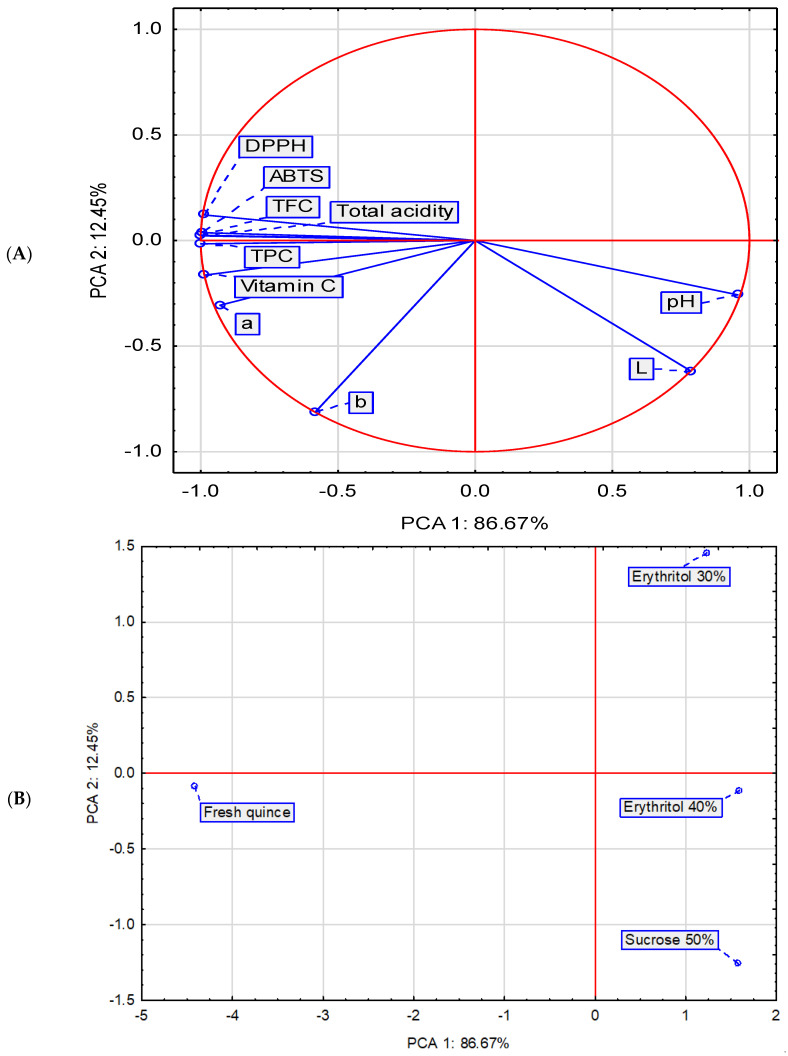
Biplot based on first two principal component axes for nutritional value of osmotically dehydrated fruits (**A**) and distribution of osmotic dehydration method based on the first two components obtained from principal component analysis (**B**).

Based on the selected parameters, a principal component analysis (PCA) was also performed, confirming the physicochemical distinctiveness of the evaluated material (fresh and osmotically dehydrated fruits) (Figure 4). The most significant difference is the pronounced reduction in the quality of the material obtained through osmotic dehydration, regardless of the solution used (Figure 4). Fresh Japanese quince exhibits significantly more favorable physicochemical properties than fruits subjected to osmotic dehydration, with the exception pH and L* parameters. Osmo-dehydrated fruits, particularly those treated with a 50% sucrose solution, exhibited a high L* value. Fruits osmotically dehydrated with a 50% sucrose solution and a 40% erythritol solution also exhibited a high pH value, although it was slightly lower than that of the material dehydrated with a 30% erythritol solution. Osmotic dehydration of quince fruits using a 30% erythritol solution resulted in a product with slightly higher values for parameters such as antioxidant activity (as measured by both DPPH and ABTS assays), total flavonoid content, and total phenolic content, but slightly lower values for vitamin C and the color parameters a* and b*. Furthermore, no correlation was found between the parameters L* and a*, as well as L* and b*, while a negative correlation was confirmed between pH, on the on hand, and DPPH and ABTS TFC and TPC, and total acidity, on the other.

## 3. Materials and Methods

### 3.1. Chemicals

Methanol, sodium carbonate, and 2,6-dichlorophenolindophenol (DCPIP) were purchased from POCh (Gliwice, Poland). Aluminum chloride, sodium nitrate, sodium hydroxide, ascorbic acid, oxalic acid, sucrose, glucose, and fructose were obtained from Chempur (Piekary Śląskie, Poland). Acetonitryle, Folin–Ciocalteu reagent, 2,2-diphenyl-1-picrylhyrazyl radical (DPPH), 2,2′-azino-bis(3-ethylbenzothiazoline-6-sulfonic acid (ABTS), gallic acid, 6-hydroxy-2,5,7,8-tetramethylchroman-2-carboxylic acid (Trolox), and quercetin were purchased from Sigma-Aldrich Chemie (Steinheim, Germany).

### 3.2. Materials

Fresh Japanese quince fruits were collected at processing maturity from local crops in Kraków (Poland). The samples were stored under refrigerated conditions (6 ± 1 °C) until the analyses were performed. Before the osmo-dehydration process, the quince fruits were washed and quartered, and the seed cores were removed. 

### 3.3. Osmotic Dehydration and Kinetic Parameters of the Process

To determine the optimal processing condition of the OD process, an analysis of the kinetics parameters water loss (WL), mass loss (ML), and solids gain (SG) was conducted. 

The osmotic dehydration process followed the procedure described by Kowalska et al. [50], with modifications. Quince fruits were placed in ground-glass conical flasks and soaked in aqueous osmotic solutions of erythritol (Intenson, Karczew, Poland) at concentrations of 20%, 30%, and 40%. To compare the kinetics of OD, a 50% sucrose solution (Pfeifer & Langen, Poznań, Poland) was used as a control (commonly used in food industry). The weight ratio of osmotic medium and fruit samples was 4:1. The OD process was carried out for 5, 15, 30, 45, 60, 90, and 120 min in a water bath with shaking (210 cycles/min) at temperatures of 20, 30, and 40 °C. Afterwards, the dehydrated samples were removed from the osmotic solution, rinsed twice with distilled water, and dried on filter paper. The kinetic parameters of all samples were calculated at different times (τ) according to the following formulas [51]:

(1) $$WL=\frac{m_{0}{\times (100-dm}_{0})-m_{\tau }\times (100-{dm}_{\tau })}{m_{0}\times {dm}_{0}}\;[g\;H_{2}O/g\;i.d.m.]$$

(2) $$ML=\frac{m_{0}-m_{\tau }}{m_{0}}\times 100\;[\%]$$

(3) $$SG=\frac{m_{\tau }\times {dm}_{\tau }-m_{0}\times {dm}_{0}}{m_{0}\times {dm}_{0}}\;[g\;d.m./g\;i.d.m.]$$

where *m*_0_—initial mass of fresh sample, *m*_τ_—mass of sample after time τ of OD, *dm*_0_—initial dry matter of sample, *dm*_τ_—dry matter of sample after time τ of OD, i. d. m.—initial dry matter. 

Based on the obtained kinetic parameters, quince fruits dehydrated in three different solutions (30% erythritol, 40% erythritol, and 50% sucrose) at a temperature of 40 °C were selected for further analysis.

### 3.4. Determination of Physicochemical Properties

The dry matter content in fresh and osmotically dehydrated fruits was determined by oven drying, initially at 70 °C by 1 h and then at 105 °C until a constant weight was achieved. pH values were determined by the potentiometric method. Total acidity (expressed as citric acid) was determined by titration with sodium hydroxide to the endpoint indicated by a phenolphthalein indicator [52]. 

### 3.5. Determination of Sugar Profile

Sugar extraction was performed by treating ground quince fruit with distilled water in an ultrasonic cleaner at 40 °C for 45 min. The resulting solution was clarified using Carrez reagents I and II and then filtered. The obtained extracts were purified using membrane filters (0.45 μm pore size) directly prior to HPLC analysis. The sugar content (fructose, glucose, sucrose, and erythritol) in the sample was determined by high-performance liquid chromatography (HPLC) with refractometric detection (LaChrom, Merck, Hitachi, Chiyoda, Japan), according to the method described by Bogdanov et al. [53]. A mixture of acetonitrile and water (80:20, *v*/*v*) was used as the mobile phase with a flow rate of 1 mL/min. The samples were separated on a Purospher Star NH2 column (Merck, Darmstadt, Germany) (250 × 4 mm, particle size 5 µm) equipped with a precolumn at 30 °C. Identification of individual saccharides was based on comparison with standards.

### 3.6. Procedure of Methanolic Extraction

The methanolic extraction was performed following the procedure described by Khattak et al. [54]. A quantity of 5 g of samples was extracted for 20 min by shaking with 15 mL of methanol (99.8%) in a screw-capped tube. Extractions were carried out three times. The combined extracts were centrifuged at 10,000 rpm for 15 min and then stored in a fridge (6  ±  1 °C) until further analysis. 

### 3.7. Determination of Total Phenolic Content

The total phenolic content (TPC) of methanolic quince fruit extracts was measured using Folin–Ciocalteu reagent, following the method described by Ainsworth and Gillespie [55]. A quantity of 0.1 mL of the extract was mixed with 2.5 mL of 0.2 M Folin–Ciocalteu reagent. After 5 min, 2 mL of 7.5% sodium carbonate solution was added, and the mixture was incubated for 2 h at room temperature. The absorbance was measured at 760 nm using a UV/VIS V-530 spectrophotometer (Jasco, Tokyo, Japan). The TPC results are expressed as milligrams of gallic acid equivalents per gram of dry matter of quince fruits.

### 3.8. Determination of Total Flavonoid Content

The total flavonoid content in quince fruit was determined using a spectrophotometric procedure described by Boateng et al. [56]. A 0.1 mL aliquot of the methanolic quince fruit extract was mixed with 4 mL of deionized water and 0.3 mL of sodium nitrate solution (15 g/100 mL). Then, 0.3 mL of aluminum chloride methanolic solution (10 g/100 mL) and 4 mL of sodium hydroxide solution (4 g/100 mL) were added, and the entire sample was diluted with deionized water to a final volume of 10 mL. After 15 min of incubation, the absorbance of the sample was measured at 510 nm. The total flavonoid content in the extracts is expressed as mg of quercetin equivalents (QE) per gram of dry matter of quince fruits.

### 3.9. Determination of Vitamin C Content

The vitamin C content was determined using the 2,6-dichlorophenolindophenol (DCPIP) titration method, following the AOAC Official Method [52]. The vitamin C content is expressed as milligrams of ascorbic acid per gram of dry matter of quince fruits.

### 3.10. Determination of DPPH Radical-Scavenging Activity

The DPPH radical-scavenging activity was measured according to the procedure described by Larrauri et al. [57]. An aliquot of 0.1 mL of the quince fruit extract was mixed with 3.9 mL of a methanolic DPPH solution (0.1 mM). After 15 min of incubation, the absorbance of the sample was measured at 515 nm using a UV/VIS V-530 spectrophotometer (Jasco, Tokyo, Japan). The antioxidant activity (AA) against DPPH is expressed as Trolox equivalents in µM per gram of dry matter of quince fruits.

### 3.11. Determination of ABTS Cation Radical-Scavenging Activity

The determination of ABTS cation radical-scavenging activity, based on the reduction of ABTS·+ by the methanolic extract of quince fruit, was carried out according to the method described by Baltrušaitytė et al. [58]. An aliquot of 0.1 mL of the methanolic extract was mixed with 6 mL of ABTS cation radical solution and incubated for 30 min. Afterward, the absorbance of the sample was measured spectrophotometrically at 734 nm. The antioxidant activity (AA) against ABTS is expressed as Trolox equivalents in µM per gram of dry matter of quince fruits.

### 3.12. Color Parameters

The color parameters of the samples were measured using a Color i5 spectrophotometer (X-Rite, Grand Rapids, MI, USA). The following settings were applied: measuring geometry d/8°, illuminant D65, and a 10° observer angle. The color data are expressed using the CIE Lab* system, with L* representing lightness, a* indicating redness, and b* indicating yellowness. Additionally, the total color differences (ΔE) were calculated according to the formula provided by Wojdyło et al. [59]:

$$\Delta E=\sqrt{{\left(\Delta L^{*}\right)}^{2}+{\left(\Delta a^{*}\right)}^{2}+{{(\Delta b}^{*})}^{2}}$$

where ΔL*, Δa*, and Δb* represent the differences in the mean L*, a*, and b* parameters, respectively, between fresh and osmo-dehydrated quince fruit.

### 3.13. Statistical Analysis

An analysis of variance (ANOVA) and principal component analysis (PCA) were carried out using the STATISTICA v13.30 software (TIBCO Software Inc., Palo Alto, CA, USA). Before ANOVA was conducted, the homogeneity of variances was checked (Levene’s test). Tukey’s Honestly Significant Difference (HSD) at *p* = 0.05 was used to find the differences between means. The means denoted by different letters differed statistically. The kinetics of the osmotic dehydration process were described using the simplest possible models, transitioning to nonlinear (curvilinear) models in cases where the linear model (straight-line) provided an insufficient fit, as indicated by a low coefficient of determination.

## 4. Conclusions

Osmotic dehydration, regardless of the osmotic agent used, caused changes in the physicochemical and antioxidant properties of fresh Japanese quince fruits. The OD process mainly affected water loss, the sugar profile, and the reduction in bioactive compounds in the fruit tissue. Osmotic dehydration in erythritol solutions leads to a partial replacement of the sugars (glucose and fructose) in Japanese quince fruits with polyol. Although the OD process with 40% erythritol solution was characterized by the most effective kinetics of the process among all applied osmotic agents, osmotically dehydrated fruits in these solutions simultaneously exhibited the most substantial alterations in physicochemical and antioxidant properties compared to fresh fruits. Notably, Japanese quince fruits osmotically dehydrated in 30% erythritol solution were characterized by higher contents of total phenolics, flavonoids, and vitamin C, as well as antioxidant activity, compared to those subjected to 50% sucrose solution, despite showing similar dehydration kinetics. The osmotic dehydration process also induced changes in the color parameters of the fruit tissue, though to a lesser extent when erythritol was used as the osmotic agent. A correlation between pH values and the L* parameter was confirmed, with the highest L* value observed in fruits osmotically dehydrated using the sucrose solution. In conclusion, erythritol, particularly at lower concentrations (30%), presents a viable alternative osmotic agent in fruit and vegetable osmo-dehydration. It offers health benefits (low-calorie and with a low glycemic index), along with potentially more energy-efficient and sustainable production, making it an attractive option for the fruit processing industry. However, its application faces certain challenges, such as the higher cost of erythritol compared to sucrose, as well as the need to optimize its concentration and dehydration times for various fruits.

## Figures and Tables

**Table 1:** Kinetics of osmotic dehydration process of Japanese quince fruits.

Factor	Factor Level	Water Loss [g H_2_O/g i.d.m.] ^1^	Mass Loss [%]	Solid Gain [g d.m./g i.d.m.]
Type of osmotic solution	Erythritol 20%	1.340 a	14.01 a	0.216 b
	Erythritol 30%	1.597 c	15.54 b	0.345 c
	Erythritol 40%	2.107 d	20.17 d	0.480 d
	Sucrose 50% (Control)	1.566 b	17.33 c	0.174 a
Temperature [°C]	20	1.416 a	14.36 a	0.266 a
	30	1.672 b	17.08 b	0.294 b
	40	1.869 c	18.86 c	0.351 c
Time [min]	5	0.778 a	8.81 a	0.077 a
	15	1.082 b	11.29 b	0.172 b
	30	1.443 c	14.71 c	0.250 c
	45	1.663 d	16.67 d	0.314 d
	60	1.930 e	19.45 e	0.373 e
	90	2.220 f	22.34 f	0.431 f
	120	2.451 g	24.08 g	0.510 g

Within columns and factors, values subscribed by the same small letters did not differ significantly at *p* = 0.05; ^1^ i. d. m—initial dry matter.

**Table 2:** Dry matter, total acidity, pH, and sugar profile of fresh and osmotically dehydrated Japanese quince fruits.

Parameters	Fresh Quince	Erythritol 30%	Erythritol 40%	Sucrose 50%
Dry matter [%]	12.39 d	28.89 b	35.35 a	23.69 c
Total acidity [%]	4.27 a	1.72 b	1.60 c	1.47 d
pH	2.93 c	3.11 b	3.13 b	3.19 a
Fructose [g/100 g d.m.]	9.23 a	1.07 c	1.17 c	7.63 b
Glucose [g/100 g d.m.]	11.87 a	1.61 c	1.47 c	7.67 b
Sucrose [g/100 g d.m.]	b.d.	b.d.	b.d.	39.02
Erythritol [g/100 g d.m.]	b.d.	40.21 b	60.43 a	b.d.
Total sugar [g/100 g d.m.]	21.09 d	42.90 c	63.07 a	54.33 b

Within rows, values denoted by the same small letters did not differ significantly at *p* = 0.05. b.d. – below detection limit.

**Table 3:** Total phenolic content (TPC), total flavonoid content (TFC), vitamin C content, and antioxidant activity in fresh and osmotically dehydrated Japanese quince fruits.

Sample	TPC [mg/g d.m.]	TFC [mg/g d.m.]	Vitamin C [mg/g d.m.]	DPPH [µM TE/g d.m.]	ABTS [µM TE/g d.m.]
Fresh quince	228.63 a	28.41 a	4.81 a	5.54 a	14.71 a
Erythritol 30%	47.69 b	11.20 b	0.67 c	2.27 b	5.96 b
Erythritol 40%	34.08 d	8.40 d	0.72 c	1.25 c	4.38 d
Sucrose 50%	40.29 c	9.77 c	1.25 b	1.50 c	5.04 c

Within columns, values denoted by the same small letters did not differ significantly at *p* = 0.05.

**Table 4:** Color parameters of fresh and osmotically dehydrated Japanese quince fruits.

Sample	L*	a*	b*	ΔE
Fresh quince	47.92 d	2.37 a	22.71 a	Standard
Erythritol 30%	51.91 c	0.36 c	18.49 c	6.17 c
Erythritol 40%	57.79 b	0.98 b	20.37 b	10.24 b
Sucrose 50%	61.66 a	0.87 b	22.15 a	13.83 a

Within columns, values denoted by the same small letters did not differ significantly at *p* = 0.05.

## Data Availability

The data presented in this study are available on request from the corresponding author.
